# Regulation of Blood Flow in the Cerebral Posterior Circulation by Parasympathetic Nerve Fibers: Physiological Background and Possible Clinical Implications in Patients With Vertebrobasilar Stroke

**DOI:** 10.3389/fneur.2021.660373

**Published:** 2021-10-29

**Authors:** Arturo Tamayo, Timo Siepmann

**Affiliations:** ^1^The Max Rady Faculty of Health Sciences, Department of Medicine, Section of Neurology, WRHA, Winnipeg and Brandon Regional Health Centre, University of Manitoba, Winnipeg, MB, Canada; ^2^Department of Health Care Sciences, Center for Clinical Research and Management Education, Dresden International University, Dresden, Germany; ^3^Department of Neurology, University Hospital Carl Gustav Carus, Technische Universität Dresden, Dresden, Germany

**Keywords:** vertebrobasilar system, ischemic stroke, autonomic system, parasympathetic system, sympathetic system, autoregulation, nitric oxide, neurovascular

## Abstract

Posterior circulation involves the vertebrobasilar arteries, which supply oxygen and glucose to vital human brainstem structures and other areas. This complex circulatory- perfusion system is not homogenous throughout the day; rather, its hemodynamic changes rely on physiological demands, ensuring brainstem perfusion. This dynamic autoregulatory pattern maintains cerebral perfusion during blood pressure changes. Accumulative evidence suggests that activity within the autonomic nervous system is involved in the regulation of cerebral blood flow. Neither the sympathetic nor parasympathetic nervous systems work independently. Functional studies have shown a tight and complicated cross talk between these systems. In pathological processes where sympathetic stimulation is present, systemic vasoconstriction is followed, representing the most important CNS parasympathetic trigger that will promote local vasodilation. Stroke is a clear example of this process. The posterior circulation is affected in 30% of strokes, causing high morbidity and mortality outcomes. Currently, the management of ischemic stroke is focused on thrombolytic treatment and endovascular thrombectomy within an overall tight 4.5 to 6 h ischemic time window. Therefore, the autonomic nervous system could represent a potential therapeutic target to modulate reperfusion after cerebral ischemia through vasodilation, which could potentially decrease infarct size and increase the thrombolytic therapeutic ischemic window. In addition, shifting the autonomic nervous system balance toward its parasympathetic branch has shown to enhance neurogenesis and decrease local inflammation. Regretfully, the vast majority of animal models and human research on neuromodulation during brain ischemia have been focused on anterior circulation with disappointing results. In addition, the source of parasympathetic inputs in the vertebrobasilar system in humans is poorly understood, substantiating a gap and controversy in this area. Here, we reviewed current available literature regarding the parasympathetic vascular function and challenges of its stimulation in the vertebrobasilar system.

## Introduction

The importance of the vertebrobasilar system (VBS) relies on the structures that are supplied (such as cerebellum, occipital lobes, and brainstem). The latter is the most important of them from a biological point of view, as it is critical for life functions. The brainstem regulates respiratory and cardiac functions, consciousness, and sleep cycles. It also plays an important role in sensory and motor functions (1, 2). Therefore, its perfusion needs to be extremely well regulated. This hemodynamic success is achieved through an active autonomic and neural system composed of the sympathetic and parasympathetic system. During extreme physiological and pathological processes, these systems are activated with the aim to prioritize cerebral blood flow to the VBS to keep its functions intact (3). In recent years, molecular biology, complex animal models, and functional imaging techniques have shown the importance of the sympathetic/parasympathetic balance function and how vascular pathology can disturb normal perfusion.

Ischemic stroke affecting the posterior circulation can have devastating consequences with high morbidity and mortality. Therefore, under a hypersympathetic state, the role of parasympathetic regulation is critical in preserving posterior circulation function (1). Thus, parasympathetic modulation in ischemia could represent a promising target for neuroprotective strategies to improve the effects of acute treatments for vertebrobasilar stroke, such as endovascular therapy and thrombolysis. However, the vast majority of animal models and human research on neuromodulation during brain ischemia have focused on anterior circulation with disappointing results. In addition, the source of parasympathetic input in the VBS in humans is poorly understood, substantiating a gap and controversy in this area. Here, we reviewed current available literature regarding the parasympathetic vascular function, its stimulation challenges, and the potential effects in posterior circulation stroke.

## Methodology

Due to the broad spectrum of topics reviewed, the authors searched medical reference databases (PubMed, EMBASE, Ovid, and Google scholar) according to assigned subtopics which, in our opinion, are critical for understanding the current topic. Anatomical references were obtained through medical textbooks as they have not changed.

### Anatomy of the Posterior Circulation and Relationships With Autonomic Structures

From its origin, the posterior circulation has critical relationships with autonomic centers which play important roles in perfusion regulation through vasodilation and constriction.

The vertebral arteries (VAs) in their V1 portion (extraosseous or pre-foraminal segment), from their origin up to the entry into the initial foramen transversarium (C6-7), are related posteriorly with rami of spinal nerves C7 and C8 and the inferior cervical ganglion. Anteromedially, it is closely related to the middle cervical ganglion (2).

In their V2 segment (foraminal), considered from C6 to C1-2., they are accompanied by sympathetic nerves as well (2).

The V3 segment (extraspinal), from C2-1 to the foramen magnum, has had no clear autonomic relationships identified. The V4 segment enters the dura mater and it is the intracranial portion of the VAs. In this segment, the roots of the hypoglossal nerve (CN XII) ascend posteriorly (1, 2).

The posterior inferior cerebellar arteries (PICA) mostly originate from the vertebral arteries traveling just above hypoglossal nerve anteriorly and just inferior to the glossopharyngeal (IX), vagus (X), and XII cranial nerves along the posterolateral medullary surface. They also supply the caudal region of the IV ventricle where the “area postrema” is located, receiving input from the (X and XII CN), aortic depressor nerves, and carotid sinuses. The area postrema (AP) also has important efferences to the locus coeruleus and tractus solitarius. The role of AP in autonomic dysfunction is clear and is not only related to vomiting. Recent evidence suggests a vasopressive response via vasopressin and angiotensin II AP receptors with sympathetic response (4, 5).

Rostrally, the BA bifurcates into the posterior cerebral arteries (PCAs) (1, 2). At the mid portion of the BA, the anterior inferior cerebellar arteries (AICAs) arise. Proximal AICA's perforating arteries supply the brainstem through branches to the internal auditory canal and the labyrinth. The rostral trunk of AICA supplies the cerebellopontine fissure and the petrosal portion of the cerebellum. The inferior trunk supplies the more inferior aspect of the petrosal surface of the cerebellum, the flocculus, and forth-ventricular choroid plexus.

Distally, the SCAs arise from the BA (3). The SCA divides into the rostral trunk that supplies the vermis and adjacent paramedian upper cerebellar hemispheres and a caudal trunk that supplies the remaining superior cerebellar hemispheres, both superiorly (tentorial surface) and anteriorly (petrosal surface). Therefore, it perfuses the superior cerebellar hemisphere and vermis and deep cerebellar white matter (3). In the 1990's, prominent research in mid cerebellar lesioned rodents demonstrated freezing motor posture abnormalities toward frightening stimuli with autonomic output dysfunction (blood pressure control and respiration rate). This has been documented in humans who have suffered medial cerebellar lesions and whose autonomic response is incomplete toward external stimulus, also called autonomic abnormalities of defensive response toward fear (6).

**Figure 1:**
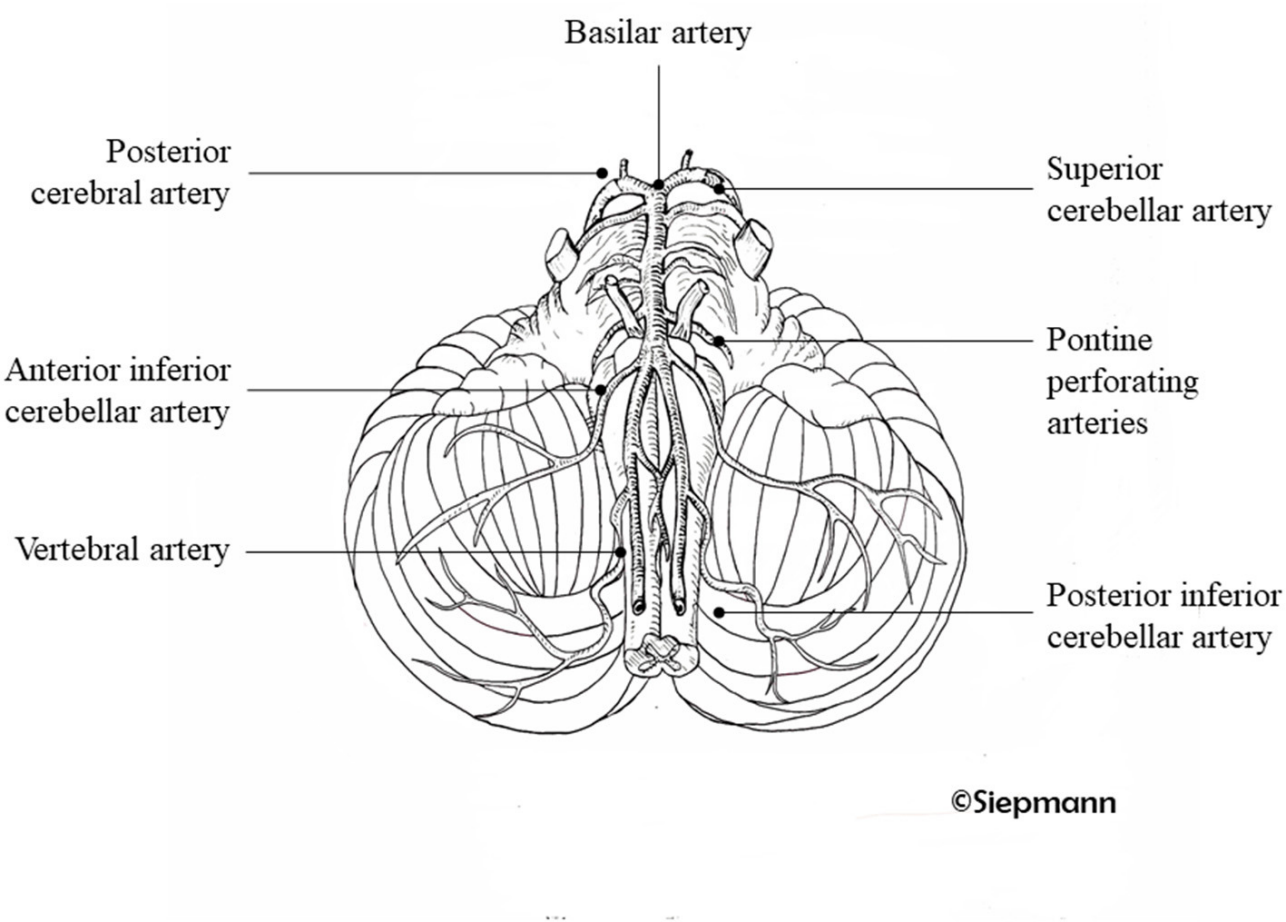
Posterior arterial circulation system. Illustration of the branches of the vertebral arteries supplying the cerebral posterior circulation system. Drawing kindly provided by Antje Siepmann.

From the terminal BA, the PCAs are where the thalamoperforating, thalamogeniculate, and peduncular perforating arteries supply the mesencephalon, diencephalon, and the choroid plexus and walls of the lateral and third ventricles and cerebral branches to the cerebral cortex and callosal splenium. (3). Vas and common branches are shown in Figure 1.

### Hemodynamics of the Posterior Circulation

The dynamics of blood flow in the posterior and anterior circulation are controlled by homeostatic mechanisms that continuously monitor and adjust to oxygen and glucose demands (7–9). The brain cannot store energetic substrates. Therefore, it requires a constant oxygen supply that can fulfill its actively changing requirements. The most important brain energy substrate is glucose. The brain oxygen requirements account for about 20% of the whole body demands in healthy adults. In children, during the first decade, the brain oxygen uptake can be as high as 50% (10). These demands are delivered at a rate of 800 ml/ min or 15–20% of the total cardiac output (CO) (9, 11).

Autoregulation plays a paramount role in modifying changes in cerebral blood flow (CBF) according to demands (9). Autoregulation, when functionally intact, allows the brain to accommodate varying levels of arterial perfusion changes within a wide range of systemic arterial pressures. When autoregulation becomes dysfunctional, the brain perfusion pressures become mostly dependent on systemic hemodynamic flow parameters (12).

Cerebral blood volume (CBV) correlates directly with CBF. Normally CBF is achieved by both cerebrovascular resistance (CVR) and cerebral perfusion pressure (CPP) (difference between systemic arterial pressure and venous back pressure).

CVR is the result or the intracranial diameter which also relies on blood viscosity. CBF and CBV will decrease as vessels constrict and vice versa (10).

The brain is a high flow low pressure with low resistance system (9, 10). This is not a new concept; in the 1800s, Monro-Kellie developed a hypothesis, describing the pressure dynamics in the relationship between brain tissue, blood and cerebrospinal fluid, and the skull. Any volume changes in any of the intracranial content will alter the intracranial pressure and dynamics with the others due to the skull's inflexibility. Normal intracranial pressures (ICP) range from 0–15 mmHg (9, 10).

CBF can be influenced by arterial blood, cerebral autoregulation, and metabolic rate through vasoneuronal coupling and nitric oxide (NO) which is an endothelium-derived relaxing factor. This is synthesized by endothelial cells from the amino acid L-arginine by the enzyme NO synthase. Among the three described NO isoforms (endothelial, neuronal, and inducible), the endothelial (eNOS) maintains blood flow by maintaining adequate vessel caliber. It has antiplatelet capabilities inhibiting platelet adhesion and aggregation, and therefore prevents thrombosis as well. Because of the antiplatelet and endothelial functions, it prevents atherosclerosis due to its vascular smooth muscle cell proliferation inhibition. In normal circumstances, there is a vasoconstriction (endothelium thromboxane and angiotensin II) - vasodilation (eNOS) intrinsic balance (13, 14).

### Cerebral Autoregulation

Cerebral autoregulation refers to a natural phenomenon in which the brain receives the same CBF in spite of moderate variations in perfusion pressure. The aim of autoregulation is to protect the brain against hypoxia and brain edema as a result of decreased perfusion pressure, and critically high arterial blood pressures respectively (10, 15). In normotensive people, effectiveness of autoregulation is seen when mean arterial pressures are below 150 and above 60 mmHg (10). Below this range, hypoperfusion starts, promoting compensatory mechanisms such as incensement of oxygen extraction from arterial beds. On the contra side, above 150 mmHg, vasoconstriction is not efficiently sustained. Initially, in this process arteries exhibit an interesting phenomenon of autoregulation with dysregulation at the same time. This is exemplified with the “sausage stringing” sign characterized by an alternating pattern of dilation with constriction within the same vessel (which are regions with residual autoregulation) (16). If CPP continues to increase, “total” dilation is seen, permitting CBF to increase passively, disrupting vascular endothelium and producing blood-brain barrier disruption leading to plasma protein extravasation (examples of this dysfunction are seen in hypertensive encephalopathy, reversible vasoconstriction syndrome, and PRES) (16).

The limits of autoregulation mentioned above vary with some physiological stimuli and certain pathologies. The vasoconstrictor effect of sympathetic activation shifts the upper and lower limits of autoregulation (17). This response is considered “protective” in response to hypertension. These thresholds are changed in chronic hypertensive patients and partially explains massive vasoconstriction seen in hyper-stimulation of the sympathetic system in pathologies such as subarachnoid hemorrhage (10, 17).

Multiple factors physiologically modify autoregulation; Brain pH changes with systemicCO2 concentrations (18). Vasodilation induced by hypercapnia stimulates cerebral blood flow (CBF). Its precise vasodilatory mechanism is not fully understood. In animal models, hypercapnia stimulates endothelial potassium ATP channels enhancing vasodilation (19); another hypothesis suggests NO as a key player in vasodilation (20). In humans, this phenomenon has been corroborated with perfusion MRI and positron emission tomography, which have shown that hypercapnia increases CBF (21–23). This response can be slightly decreased but still prominent in older populations due to atherosclerotic changes that might interfere with full vasodilation. This effect is not ambiguous, and some acute neuropsychiatric phenomena have been associated with it (24).

Hypoxia on the other hand promotes vasodilation due to hypoxic effect. Also, this response is mediated through nitric oxide and adenosine. This has been nicely investigated in climbers where rapid oxygen therapy modified vasodilation (25).

### Parasympathetic Innervation of the Vertebrobasilar Arteries

The parasympathetic innervation of intracranial arteries (IA) possess two important differences in comparison to the extracranial arterial system: The IA parasympathetic innervation is 10–40 times higher, providing a powerful vasodilatory mechanism (26, 27). Another difference is that IA are minimally or not cholinergic dependent, that is, there is an inability to produce acetylcholine (ACh) as a transmitter (28), rather, vasoactive intestinal peptide (VIP), pituitary adenylate cyclase-activating peptide (PACAP), and most importantly NO are the main co-transmitters in parasympathetic neurons which, interestingly, are all very potent vasodilators, perhaps even more so than ACh (28–30). However, it seems that NO is the main vasodilator and the rest are mostly pre-synaptic modulators for NO release (29, 30).

### Anterior Vs. Posterior Circulation Vascular Innervation

The origin of the parasympathetic innervation in posterior circulation is not fully understood and it differs from the anterior circulation. There is a common origin at the skull base and diffusely in the arachnoid space, identified as a diffuse collection of small cell groups.

In the anterior circulation the innervation is provided by fibers from the sphenopalatine ganglia (SPG), commonly known as pterygopalatine ganglia (PPG), the otic ganglia, the cavernous sinus, and the internal carotid mini-ganglia (CMG) (31, 32).

There is evidence of parasympathetic activity in the VBS but in humans the source and inputs are not fully known (33).

Animal models (34) have located preganglionic neurons projecting to the PPG, stimulating the ipsilateral superior salivatory nucleus (SSN) and specifically the greater petrosal nerve. This creates a stimulation cascade activating center in medulla oblongata (spinal trigeminal nucleus, the nucleus of the tractus solitarii or NTS, which plays an important processing role in autonomic response to hypoxia), pontine nucleus (parabrachial structures), and gigantocellular reticular nucleus and subcoeruleus region and A5 catecholaminergic cells (34, 35), paraventricular hypothalamic nuclei and the lateral pre-optic area. The bed nucleus of the stria terminalis, substantia innominate and the amygdalopiriform, the otic ganglia and CMG (33, 36) This nuclei-web enhances the importance of the SSN vasodilatory effect and the regions that may influence it (33).

### Neuromodulation of the Posterior Circulation

The most important vasodilator neurotransmitter is NO, mostly clustered in the basilar artery. NO possesses a half-life of 5–15 seconds and is produced through cholinergic neurons and VIPergic neurons (37). Endothelial cells, astrocytes, neurons, and immune cells are other sources of NO that contribute to VB dilation (33, 38–40).

Endothelial and smooth muscle cells express cholinergic receptors. However, the effectiveness of these receptors is not through acetylcholine, rather it is through muscarinic (M) and nicotinic (N) receptors (33, 41). There have been four muscarinic and one nicotinic type of receptors identified. NO-dependent vasodilation is achieved when Ach activates M and N receptors (42, 43). M1and 3 receptors mediate the NO-induced vasodilation in humans. M1 is mostly seen in the basilar artery (43). Their affinity for Ach is lower in comparison to M3 receptors that show high Ach affinity and requires high concentrations of Ach for activation. In the presence of functional endothelium M3 receptors, the activity of M1 is not minimal. This explains the dominance of M3 in intracranial circulation playing the major role in vasodilation (33, 44). In animals' M2 receptors, function is vasoconstrictive through inhibition of NO. These receptors have not been found in humans (43). The M5 receptor is also an M-type receptor the function of which is not well understood and some studies have proposed this to be similar to M3 in animals. This is, again, not confirmed in humans (45).

The vasodilator function of the vasoactive intestinal peptide (VIP) is not fully understood. VIP co-localizes with cholinergic acetyltransferase in some parasympathetic nerves and has been isolated mostly in the basilar artery (46, 47). The vasodilator effect of VIP could be through NO via the natriuretic peptide clearance receptors (NPRC) (47).

The final neuropeptides that play a role in vasodilation (but with weaker properties) are the human peptide histidine methionine and pituitary adenylate cyclase activating peptide (48, 49) which, due to poor vasodilatory function, will not be discussed further in this paper. The nicotinic receptors will be discussed shortly.

### Parasympathetic and Sympathetic Inter-communication

It is not infrequently observed that parasympathetic and sympathetic nerves are interlaced in the same perineural sheath innervating cerebral arteries. Functional studies in animals' basilar arteries have shown poor vascular response to Ach and noradrenaline. This is not seen with adrenergic nerves which express nicotinic Ach receptors that once activated enhances intracellular calcium influx. Intracellular calcium then stimulates the release of noradrenaline, triggering vasodilation via β-2 adrenergic receptors. These receptors are located within the Ach-nitrergic fibers where NO is stored and released once NO and Ach are released. Modulating NO, pre-junctional M2 receptors are stimulated simultaneously with NO and Ach, inhibiting further NO synthesis (negative feedback phenomena). During β2 adrenergic stimulation, vasoconstrictions occur no more due to paucity of α-adrenoceptors (50, 51). In face of increased sympathetic drive, the expected response in the VB arteries would be dilation rather than constriction. This response guarantees, in normal circumstances, optimal CNS perfusion during systemic hypoperfusion due to adrenergic constriction (52).

### Parasympathetic Pathophysiology of Stroke

Stroke is a neurovascular emergency secondary to intracranial ischemia or hemorrhage that leads to a specific neurological dysfunction related to the affected area. Unlike TIA (transient ischemic attack), the deficit in stroke usually persists >24 hours (53, 54). Approximately 30% of these strokes are located in the VBS (54, 55). Ischemia is secondary to a vascular occlusion. In the posterior circulation, etiological factors to consider are local thrombotic events in the VBA (30%), dissections or embolism, usually cardioembolic (40%) and small vessel related. In 30% of patients an etiological factor might not be found (54). Therefore, occlusive atherosclerosis is a major stroke risk factor supporting the role of hemodynamic insufficiency in the posterior circulation. This has been recently supported in the Vertebrobasilar Flow Evaluation and Risk of Transient Ischemic Attack and Stroke (VERiTAS) study, which monitored the hemodynamic status in posterior circulation with measurements of large cerebral vessel flow. This was achieved using phase-contrast quantitative magnetic resonance angiography (QMRA), which is considered a very reliable surrogate for vascular hemodynamic status (for further description of methodology, readers are encouraged to search for more specialized imaging literature). The VERiTAS study demonstrated with QMRA that hemodynamic compromised posterior circulation is a robust predictor for stroke, reaching a 4-fold higher risk over 24 months (56, 57) and the potential for both improvement and worsening relies on hemodynamic shifting over time. That is, if hypoperfusion continues, the stroke risk increases over two years and vice-versa. (58). In the acute setting, the aim of the parasympathetic vasodilatory function is to preserve cerebral blood flow, decrease ischemic core, and promote hemodynamic hypoperfusion which can lead to ischemic core extension and stroke recurrence (59, 60).

### Therapeutic Activation of Parasympathetic System in Stroke Patients

#### Vagus Nerve Stimulation (VNS)

This can be summarized in four different mechanisms: effect on neurotransmitters, anti-inflammatory pathways, modulation of cerebral blood flow, and neurogenesis enhancement.

#### Neurotransmitters

Decreasing extracellular glutamate decreases the stimulation of N-methyl-D-aspartate receptors,VNS decreases cerebral ischemic penumbra and thus, infarct size, promoting less neurological deficit impairment (59, 61). Speculations regarding other mechanisms of action (59) suggest that VNS also stimulates norepinephrine and 5-HT which decreases extracellular glutamate as well (59). In humans, most studies have been focused on chronic stroke aiming to determine safety and improvement of rehabilitation outcomes. This strategy has shown to be promising in acute ischemic strokes (62).

#### Cholinergic Anti-inflammatory Pathway

After ischemic stroke, tissue inflammation worsens outcomes (63). Shortly after ischemia, resident microglia are activated and accumulate at the lesion site and penumbra (peaks in 72 h). Upon activation, microglia undergoing proliferating factors are released, among them chemokines and cytokines, such as tumor necrosis factor (TNF)-α, interleukin (IL)-1β, and neurovascular proteases such as matrix metalloproteinase (MMP-9), are prominent, generating reactive oxygen and nitrogen species (ROS), via NADPH oxidase (nicotinamide adenine dinucleotide phosphate) (64, 65). The cholinergic anti-inflammatory pathway suppresses inflammation. This effect is through inhibition of cytokines expression and therefore inflammation (factorn-κB pathway) (66).

The vagus nerve represents the peripheral efferent output from a complex cortical cholinergic (Ach) system. The main properties are to reduce microglia inflammation through activation of the nAChRα7 receptors. These anti-inflammatory properties are activated through VNS (67). Parasympathetic stimulation also decreases the systemic inflammatory response (C-reactive protein among others) which can potentially worsen stroke outcomes. Pharmacologically, Ghrelin stimulates vagus-related nerve-mediated anti-inflammatory markers including cytokines and tumor necrosis α. (59).

#### CBF Modulation by Parasympathetic Stimuli

In this topic, complementing what we already discussed, SPG innervate intracranial vessels that express receptors to their nitroxidergic-cholinergic cells. Its stimulation increases cerebral blood flow through NO vasodilation, decreasing diffusion – perfusion mismatch in functional MRI studies. This effect is mostly seen in the penumbra area, avoiding further apoptosis and progression of infarct core size (68). Another important effect seen with SPG stimulation is the enhancement of the blood brain barrier permeability, which could be beneficial when using a neuroprotective agent, however, on the other hand, BBB permeability has shown risk of hemorrhage during thrombolytic therapy (69). In the authors' opinion, this lack of permeability and perfusion could explain why many neuroprotectant agents have failed to provide benefit. These findings could open new areas of interest in future neuroprotective research in stroke.

#### Parasympathetic Activation Enhances Neurogenesis

Stroke promotes neural stem proliferation conditions (70). Norepinephrine, brain derived neurotrophic factor, fibroblast grow factors, and 5-HT are promoted through VNS.

Norepinephrine and 5-HT regulate extensive cholinergic innervated hippocampal neurogenesis (survival, integration of newborn neurons, and maturation) through nAChRα7 (59, 71). Thus, neurogenesis enhancement could be achieved with VNS or with nAChRα7 agonists (59).

### Therapeutic Perspectives

**Figure 2:**
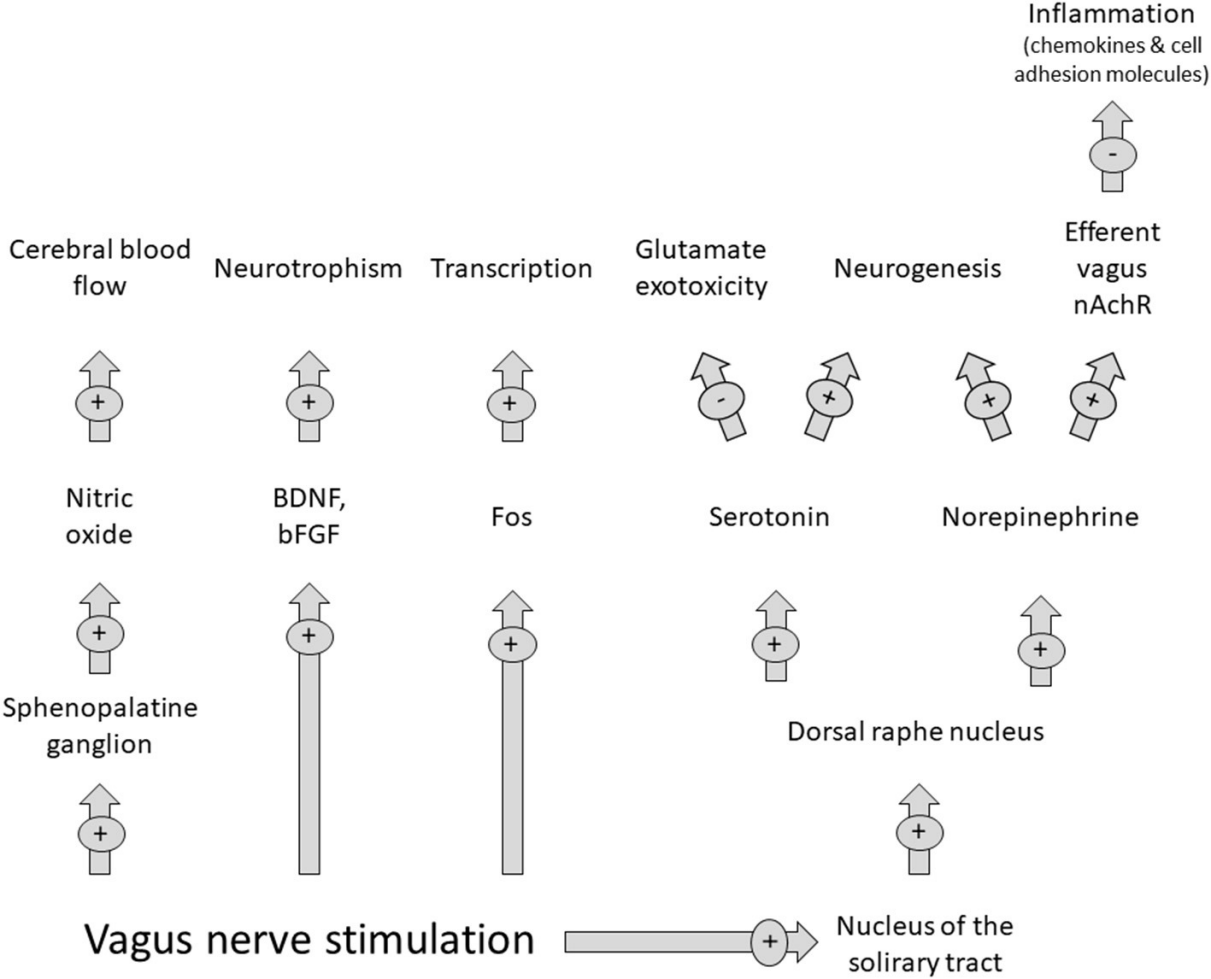
Effects of vagus nerve stimulation on the parasympathetic nervous system. Abbreviations: BDNF: brain derived neurotrophic factor; bFGF, brain fibroblast growthfactor. Modified from Cai PY. (2014) Frontiers in Neurology 10.3389/fneurol.2014.00107.

Parasympathetic stimulation through VNS is not a new concept. It has been used in other areas of neurology (epilepsy) and psychiatry (depression) (72). This is due to its ability to interfere with the ischemic stroke cascade pathophysiology through mechanisms such as norepinephrine and serotonin release. VNS has shown stroke size reduction in experimental animal models attenuating stroke volumes (72) and post stroke outcomes studied in rehabilitation only in anterior circulation strokes. Figure 2 summarizes VNS effects during stroke.

Recently, the NOVIS trial (Non-invasive Vagus nerve stimulation in acute Ischemic Stroke) protocol was published (73) and is expected to start in 2021. This will be an open label clinical trial with blinded outcome assessment in 150 anterior circulation stroke patients at the Leiden University Medical Center. However, to date no VNS trial has focused on posterior circulation.

The parasympathetic effect of sphenopalatine ganglion stimulation has been recently studied in the ImpACT-24A trial (Sphenopalatine ganglion stimulation to augment cerebral blood flow. A randomized, sham-controlled trial) (74). They randomized 253 patients in the study where 153 were included in the study treatment and 100 in the control or sham. The primary outcome was improvement in disability beyond expectations after three months, which was shown to be 49.7 vs. 40%: odds ratio, 1.48 (95% CI, 0.89–2.47) *p* = 0.13. However, a significant treatment interaction with stroke location (cortical vs. non-cortical) was noticed. In 87 patients with confirmed cortical involvement, rates of improvement beyond expectations were 50.0 vs. 27.0% odds ratio, 2.70 (95% CI, 1.08-6.73);*P* = 0.03. The final results of the trial (75) did show a good safety profile but failed to reject the null hypothesis supporting SPG.

The use of α-7 nicotinic acetylcholine receptor agonist has shown in anterior circulation stroke-induced models in animals some neuroprotective effect reducing inflammation and oxidative stress. There is a need for further studies in this regard (76).

### Facial Nerve System Stimulation

Stimulating the facial nerve directly stimulates SPG and the Otic ganglia. This produces further activation cascade only to VIP-expressing nerve fibers mostly found in the carotid arteries and anterior intracranial circulation. The final result is vasodilation. In the posterior circulation there is mild to non-VIP nerve expression and can only be found in the rostral portion of the basilar artery which is stimulated through the cavernous ganglia once the facial nerve is stimulated (77).

### Cavernous Ganglia Stimulation

Brilliant descriptions of its stimulation through the SPG have been done in animal models (78), demonstrating vasodilation in anterior and posterior circulation. The latest after stimulation follows the IV and VI CN backward to the basilar artery and tentorium cerebelli respectively. However, no clinical studies have been done in humans.

## Conclusions

Our current knowledge of the autonomic vascular system innervation is still weak. The anatomical variants in different animal species have complicated this even further. The fact of not knowing the real parasympathetic origin in posterior circulation widens this gap, as no direct neuromodulation mechanism can stimulate the posterior circulation in isolation. However, one important feature learned is that this expected “isolated” system in anterior and posterior circulation seems non-existent. Therefore, functional imaging studies are needed to determine hemodynamics in posterior circulation during traditional parasympathetic stimulation in anterior circulation.

The protective role of the parasympathetic system in cerebral arteries (under hypersympathetic stimulation) in ischemic strokes is undoubtable. We believe that most research failures in humans could have been related to the long ischemic windows with no other intervention (IV thrombolytics or mechanical thrombectomy). This could represent another venue for future research. Furthermore, the lack of success with all neuroprotectant agents could have been related to the local hypoperfusion that ischemic stroke implies per se. Therefore, and speculating, the means of proper stimulation of this system might open the doors to more options in stroke patients.

While the stimulation of this complex system has been debated (78–82), in our opinion we believe the issue to be clear. We require more strict protocols with shorter ischemic windows, not forgetting that there is an approved management practice, as this could represent, in the future, ways to optimize our acute management in these critically ill patients.

## Author Contributions

AT contributed to conception and design of the article, reviewed literature and wrote content. TS contributed to conception and design of the article, reviewed and improved manuscript content. Both authors contributed to the article and approved the submitted version.

## Funding

This review paper was part of a Master's thesis project at Dresden International University. We acknowledge support by the Open Access Publication Funds of the SLUB/TU Dresden. Dr. Siepmann received grants from the Federal German Ministry of Health, the Kurt Goldstein Institute, and the Michael J. Fox Foundation that were not related to this article.

## Conflict of Interest

The authors declare that the research was conducted in the absence of any commercial or financial relationships that could be construed as a potential conflict of interest. The reviewer DB declared a past co-authorship with one of the authors TS to the handling Editor.

## Publisher's Note

All claims expressed in this article are solely those of the authors and do not necessarily represent those of their affiliated organizations, or those of the publisher, the editors and the reviewers. Any product that may be evaluated in this article, or claim that may be made by its manufacturer, is not guaranteed or endorsed by the publisher.
